# Applying a CT texture analysis model trained with deep‐learning reconstruction images to iterative reconstruction images in pulmonary nodule diagnosis

**DOI:** 10.1002/acm2.13759

**Published:** 2022-08-23

**Authors:** Qingle Wang, Shijie Xu, Guozhi Zhang, Xingwei Zhang, Junying Gu, Shuyi Yang, Mengsu Zeng, Zhiyong Zhang

**Affiliations:** ^1^ Department of Radiology, Zhongshan Hospital Fudan University Shanghai China; ^2^ Shanghai Institute of Medical Imaging Shanghai China; ^3^ Department of Medical Imaging, Shanghai Medical College Fudan University Shanghai China; ^4^ Shanghai United Imaging Healthcare Shanghai China

**Keywords:** CT, deep learning reconstruction, pulmonary nodules, texture analysis

## Abstract

**Objective:**

To investigate the feasibility and accuracy of applying a computed tomography (CT) texture analysis model trained with deep‐learning reconstruction images to iterative reconstruction images for classifying pulmonary nodules.

**Materials and methods:**

CT images of 102 patients, with a total of 118 pulmonary nodules (52 benign, 66 malignant) were retrospectively reconstructed with a deep‐learning reconstruction (artificial intelligence iterative reconstruction [AIIR]) and a hybrid iterative reconstruction (HIR) technique. The AIIR data were divided into a training (*n* = 96) and a validation set (*n* = 22), and the HIR data were set as the test set (*n* = 118). Extracted texture features were compared using the Mann‐Whitney *U* test and *t*‐test. The diagnostic performance of the classification model was analyzed with the receiver operating characteristic curve (ROC), the area under ROC (AUC), sensitivity, specificity, and accuracy.

**Results:**

Among the obtained 68 texture features, 51 (75.0%) were not influenced by the change of reconstruction algorithm (*p* > 0.05). Forty‐four features were significantly different between benign and malignant nodules (*p* < 0.05) for the AIIR dataset, which were selected to build the classification model. The accuracy and AUC of the classification model were 92.3% and 0.91 (95% confidence interval [CI], 0.74−0.90) with the validation set, which were 80.0% and 0.80 (95% CI, 0.68−0.86) with the test set.

**Conclusion:**

With the CT texture analysis model trained with deep‐learning reconstruction (AIIR) images showing favorable diagnostic accuracy in discriminating benign and malignant pulmonary nodules, it also has certain applicability to the iterative reconstruction (HIR) images.

## 1 INTRODUCTION

Texture analysis in medical imaging plays an important role in various applications nowadays, such as distinguishing malignant from benign lesions, predicting clinical outcomes, and making the therapeutic choice for patients with nonsmall cell lung cancer.^1^ ^−^ ^5^ Texture analysis is normally conducted in three steps: (1) image segmentation, (2) features extraction and filtering, and (3) classification modeling. The texture features are generally divided into three categories: first‐order features, second‐order features, and high‐order features. First‐order features analyze the distribution of grayscale intensity histograms, such as the mean intensity, skewness, and kurtosis. Second‐order features evaluate the relations among adjacent pixels. Higher‐order features, such as coarseness and busyness, evaluate neighboring voxels.^6^ In the early research, multiple logistic regression and multivariate Cox proportional hazard regression were used to establish a predictive model.^7^, ^8^ In recent years, the application of artificial intelligence algorithms in predictive models has greatly improved the diagnostic accuracy, such as random forests^9^ and convolutional neural networks.^10^

Computed tomography (CT) is a widely used diagnostic imaging modality in oncology, and CT images are one type of the most commonly adopted data sources in current texture analysis studies. In parallel to the development of texture analysis, CT itself also undergoes continuous development and technical innovation. The most recent example is the deep‐learning‐based reconstruction,^11^, ^12^ which has proven to be another major step toward ultra‐low‐dose CT^13^ ^−^ ^15^ as compared to iterative reconstruction that prevailed over the last decade.^16^, ^17^

Texture analysis benefits from the introduction of the deeplearning‐based reconstruction technique, for its improved the image quality^18^ as well as reproducibility of texture features in general.^19^ Since texture analysis relies on the extracted feature values, it is easily affected by scanning parameters and reconstruction algorithms.^20^, ^21^ When performed on the images with higher resolution and less noise, texture analysis shows a better diagnostics performance in classifying lesions.^22^, ^23^

However, such advanced CT reconstruction algorithms may not always be available at all times and in places where last‐generation reconstruction algorithms are still in active use. This leads to an interesting question whether images obtained with relatively old reconstruction algorithm can be applied to models that are originally derived from data of the latest deep‐learning reconstruction, and if yes, how reliable it can be. In other words, re‐training would become unnecessary if the models obtained remain applicable.

The aim of this study was to investigate the feasibility and accuracy of such applicability for CT texture analysis models through a task aimed on classifying benign/malignant pulmonary nodules, which is a task frequently visited and found with the highest robustness in recent researches.^24^, ^25^ In this study, CT images were reconstructed with two different algorithms. The classification model was built using images of the latest‐generation technique, that is, the deep‐learning reconstruction, and then tested using images of the last‐generation iterative reconstruction.

## 2 METHODS AND MATERIALS

This retrospective study was performed on archival raw data of chest CT examination. This subsequent image analysis was approved by the local institutional review board, and written informed consent was waived due to the retrospective nature of this study.

### 2.1 Two generations CT reconstruction

The deep‐learning reconstruction, that is, the latest‐generation method considered in this investigation, is the artificial intelligence iterative reconstruction (AIIR) provided with the multidetector‐row uCT systems (United Imaging Healthcare). The AIIR algorithm is an improved version of the previously reported deep‐learning reconstruction.^26^ Technically, it uses a deep learning‐based denoiser to replace the regularization in the traditional model‐based iterative reconstruction (MBIR) algorithm to reduce noise, which not only ensures a substantial noise suppression and avoids the plastic feeling that is easily caused by the regularization algorithm, but also retains the consistent advantage of the MBIR algorithm, such as the absence of noise‐induced streaking artifacts, better truncation performance, and fewer cone‐beam artifacts.^27^, ^28^

**FIGURE 1:**
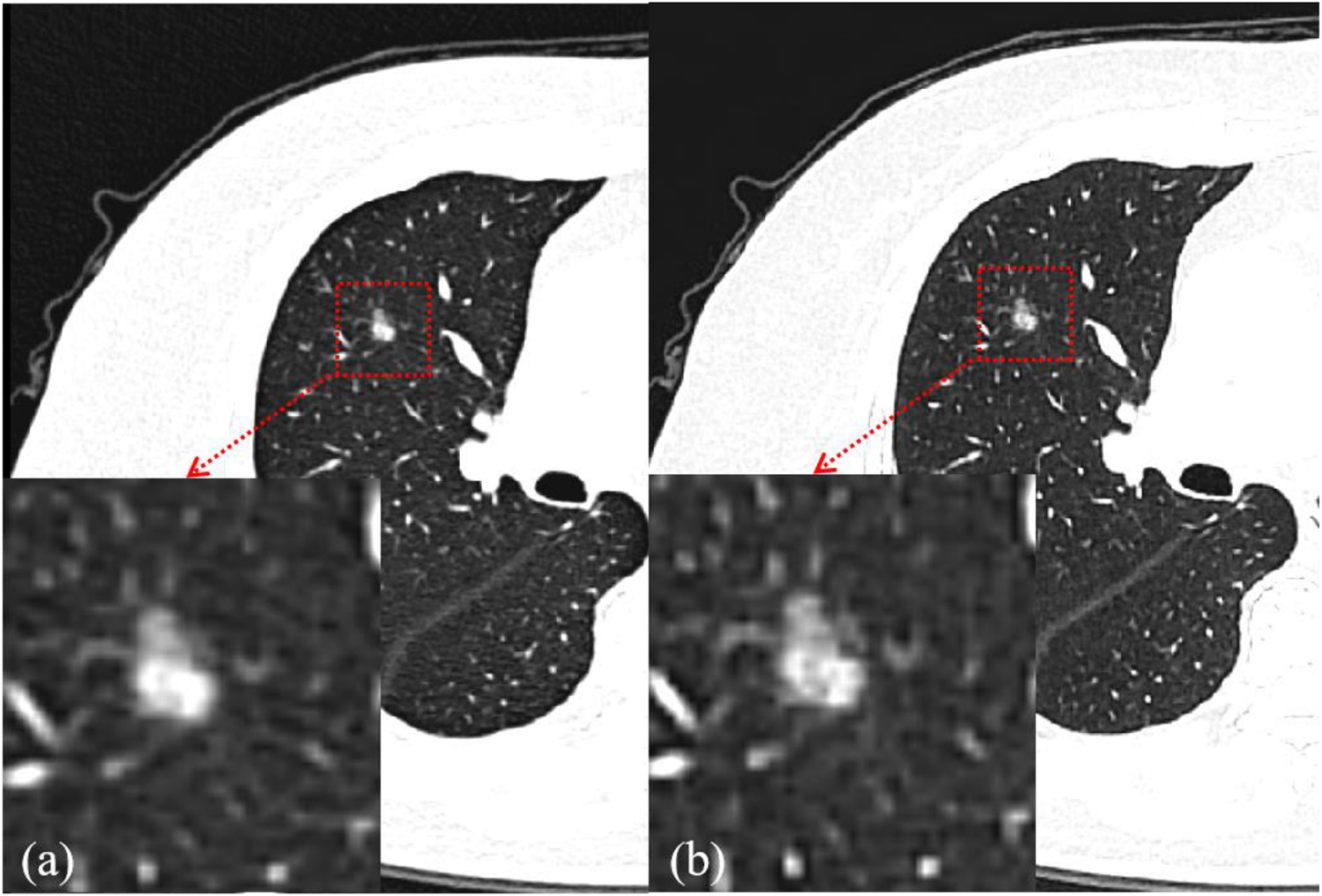
Hybrid iterative reconstruction (HIR) (a) and artificial intelligence iterative reconstruction (AIIR) (b) in routine‐dose Computed tomography (CT) performed on a 69‐year‐old woman with malignant pulmonary nodule (adenocarcinoma) in right lobe. Compared with the HIR algorithm, the contours of the nodule is clearer, the lung texture is more distinct, and the noise is less in images reconstructed by AIIR algorithm.

The last‐generation reconstruction considered in this investigation is a hybrid iterative reconstruction (HIR) technique (KARL, United Imaging Healthcare).^29^ It denoises in both the projection space and the image space. Compared to MBIR, less computing power is required by using generalized gradient descent to accelerate the reconstruction speed with fast convergence to the objective value in the optimization process. KARL and other HIR algorithms are now available with most commercial CT systems and have gained wide acceptance in various CT applications. The comparison of CT images reconstructed by two generations reconstruction is shown in Figure 1.

### 2.2 Patient data

From January 2020 to July 2021, a total of 130 patients in our hospital suspected of lung cancer who underwent chest CT examination were enrolled in this study. In total, 89 patients were with pathological results, and 41 patients had diagnostic results from follow‐up CT. In order to ensure the accuracy of the classification and segmentation results, 12 patients were excluded due to poor image quality, and 16 patients were excluded because the CT images could not be reconstructed due to the corruption of the raw data. Finally, 102 patients with 118 pulmonary nodules were included. The patient characteristics were shown in Table 1. Four patients were with both benign and malignant nodules. Pulmonary nodules were divided into two groups according to the results of pathology and follow‐up data: benign (*n* = 52) and malignant group (*n* = 66).

**TABLE 1:** Patients demographics

Patient characteristics	
Number	102
Age (years)*	61.7 ± 13.7
Gender	
Male	52 (51.0%)
Female	50 (49.0%)
Type of nodule	
Benign	52 (44.1%)
Malignant	66 (55.9%)
Location	
Left lobe	58 (49.2%)
Right lobe	60 (50.8%)

* Presented as mean ± standard deviation.

### 2.3 CT acquisition and image reconstruction

All examinations were performed with a multidetector CT scanner (uCT760, United Imaging Healthcare, Shanghai, China) using the following acquisition parameters as in routine chest examination: 80 × 0.5 mm collimation, 100/120 kV by auto‐kV, tube current modulation on, 0.5 s rotation time, 0.96 spiral pitch. All images were reconstructed with the same settings using AIIR and HIR: 350 mm field of view, 512 ⋅ 512 pixel matrix, 1.0 mm slice thickness and 1.0 mm slice interval.

**FIGURE 2:**
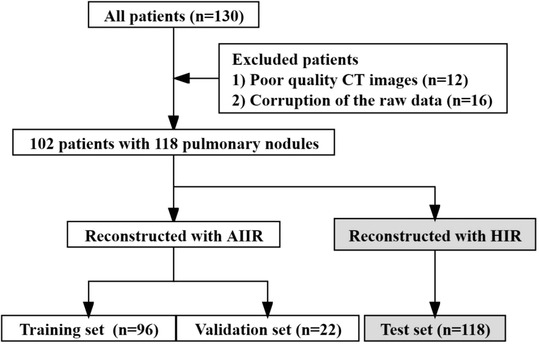
Scheme of case collection

The images reconstructed with AIIR were further separated into a training set (*n* = 96) for building the classification model, and a small validation set (*n* = 22) for self‐testing, while the ones with HIR were set as the test set (*n* = 118) in order to assess its applicability on the last‐generation iterative reconstruction images. The flow chart of the study is shown in Figure 2.

### 2.4 Nodules segmentation and extraction

**FIGURE 3:**
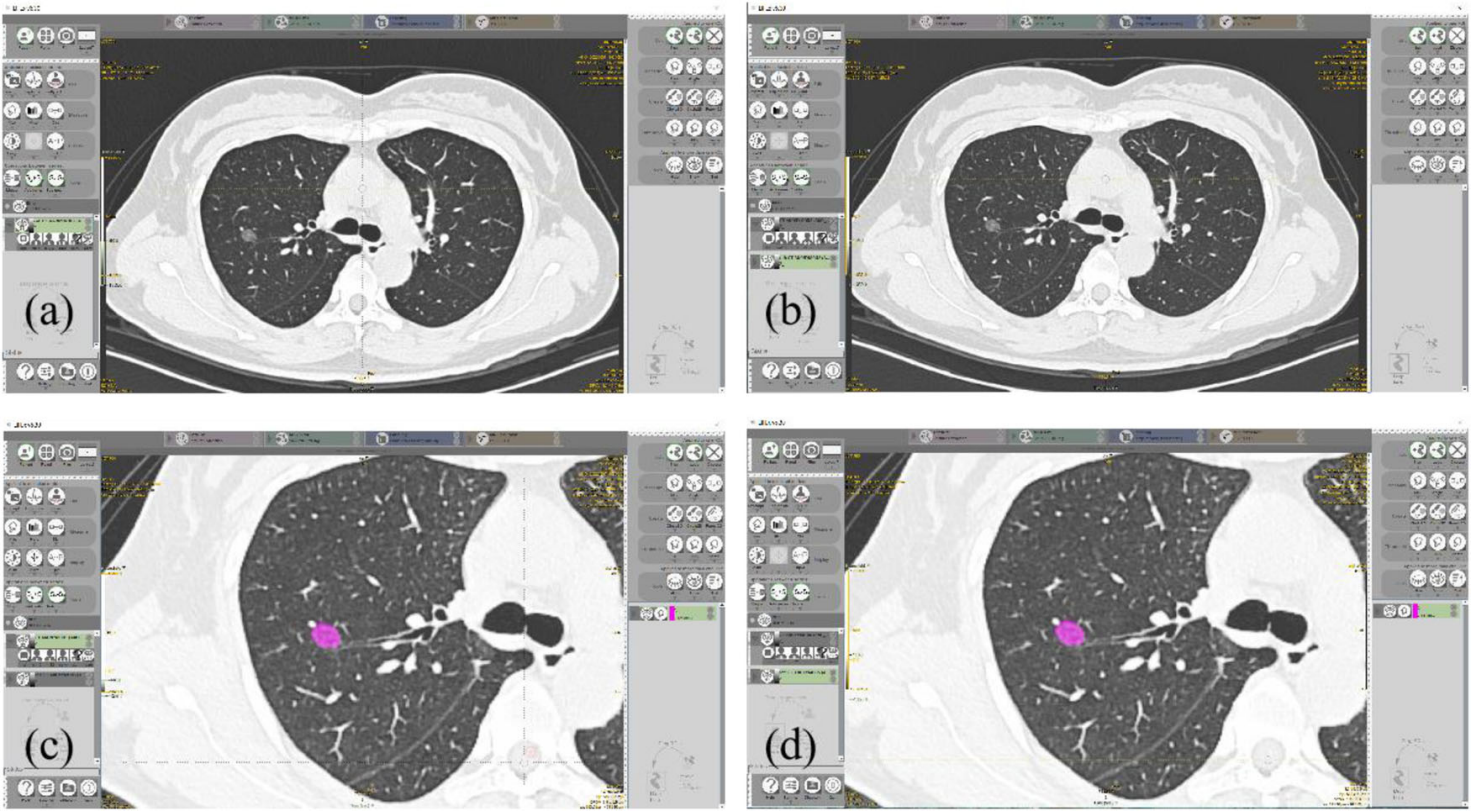
The example for segmentation. Firstly, artificial intelligence iterative reconstruction (AIIR) images were loaded into the segmentation software (a). Then, hybrid iterative reconstruction (HIR) images were loaded (b). After that, the contour of pulmonary nodule was outlined on the AIIR images manually (c). Finally, the segmentation was translated to the HIR images at the same position (d).

Features extraction was performed using the LIFEx software (www.lifexsoft.org).^30^ One radiologist with 3 years of experience in chest imaging manually outlined the lesions on CT images with a display W/L = 1000/‐600HU. As recommended in the study by Sarioglu,^2^ a total of seven categories of features were extracted: conventional features, discretized features, shape features, gray‐level co‐occurrence matrix (GLCM), gray‐level run‐length matrix (GLRLM), neighborhood gray‐level difference matrix (NGLDM), and gray‐level zone length matrix (GLZLM) features. The texture features were extracted from the AIIR images as well as the HIR images. The segmented contours were first outlined on the AIIR images and then translated onto the HIR images at the same position (Figure 3).

### 2.5 Features selection and classification modeling

Feature significantly different between benign and malignant nodules was selected as the input of the classification model. The classification model was built using the multilayer perceptron (MLP).^31^ The MLP is an artificial neural network for supervised classification and selects features at different scales to make a prediction. In addition to an input layer (44 nodes) and an output layer, there were two hidden layers (10 nodes for each layer). The activation function for hidden units was the hyperbolic tangent function, and for the output was the identity function. To evaluate the performance of the model on AIIR images, the leave‐one‐out cross validation (LOOCV) was carried out in the first place. The classification model derived from the AIIR images was then applied to the test set, that is, the classification model was built using only the features extracted from AIIR images and then tested using features extracted from HIR images. To validate the accuracy of the model itself, the AIIR images were further divided into a training group and a validation group according to a ratio of 4:1.

### 2.6 Statistical analysis

All statistical data were analyzed using SPSS 22.0 software. Continuous data were presented as means and standard deviation, when categorical variables presented as frequency counts and percentages. The distribution of variables was analyzed using the Kolmogorov‐Smirnov and Shapiro‐Wilk test. The values of features extracted from AIIR and HIR images were compared. Features in normal distribution were analyzed using the Mann–Whitney *U* test, and those not in normal distribution were analyzed using *t*‐test. A *p* < 0.05 was considered statistically significant. The diagnostic performance of each texture feature and the classification model was evaluated by the receiver operative characteristic curve (ROC) analysis. Sensitivity, specificity, negative predictive value (NPV), positive predictive value (PPV), accuracy and the area under ROC (AUC) were calculated and compared. The AUC was compared by calculating the 95% confidence interval (CI).

## 3 RESULTS

### 3.1 Comparison of extracted features between two reconstruction algorithms

In total, 68 features were obtained: 13 conventional features, 18 discretized features, 5 shape features, 7 GLCM features, 11 GLRLM features, 3 NGLDM features, and 11 GLZLM features. Among these 68 features, four features following the normal distribution were analyzed using *t*‐test, and the other 64 features not following the normal distribution were analyzed using the Mann–Whitney *U* test. Comparing the features extracted from AIIR and HIR dataset: only 25.0% (17 of 68) features were significantly influenced by the reconstruction algorithm (*p* < 0.05), while the remaining 75.0% (51 of 68) features were not, as shown in Table 2. Among the seven categories of features, only the features in the shape category were not influenced by two reconstruction algorithms, which meant that the other six categories, including conventional, discretized, GLCM, GLRLM, NGLDM, and GLZLM features, were all influenced by two reconstruction algorithms.

**TABLE 2:** The comparison of features between the two generations reconstruction

Feature		Feature		Feature	
Conventional	*p‐*Value	Discretized	*p‐*Value	GLZLM	*p‐*Value
HUmin	0.07	HUmin	0.20	SZE	0.08
HUmean	0.42	HUmean	0.43	LZE	0.83
HUstd	0.89	HUstd	0.87	LGZE	<0.001
HUmax	0.01	HUmax	0.01	HGZE	0.72
HUQ1	0.33	HUQ1	0.32	SZLGE	<0.001
HUQ2	0.52	HUQ2	0.50	SZHGE	0.92
HUQ3	0.16	HUQ3	0.15	LZLGE	0.01
HUSkewness	0.59	HUSkewness	0.56	LZHGE	0.29
HUKurtosis	0.26	HUKurtosis	0.28	GLNU	0.73
HUExcessKurtosis	0.26	HUExcessKurtosis	0.28	ZLNU	0.67
HUpeakSphere0.5 ml	0.88	HUpeakSphere0.5 ml	0.88	ZP	0.32
HUpeakSphere1 ml	0.85	HUpeakSphere1 ml	0.85	**GLRLM**	
HUcalciumAgatstonScore	0.45	HISTO_Skewness	<0.001	SRE	0.36
**GLCM**		HISTO_Kurtosis	<0.001	LRE	0.44
Homogeneity	0.12	HISTO_ExcessKurtosis	<0.001	LGRE	<0.001
Energy	0.87	HISTO_Entropy_log10	0.29	HGRE	0.50
Contrast	0.01	HISTO_Entropy_log2	0.29	SRLGE	<0.001
Correlation	<0.001	HISTO_Energy	0.05	SRHGE	0.51
Entropy_log10	0.92	**SHAPE**		LRLGE	<0.001
Entropy_log2	0.92	Volume (ml)	0.999	LRHGE	0.49
Dissimilarity	0.04	Volume (vx)	0.976	GLNU	0.62
**NGLDM**		Sphericity	0.926	RLNU	0.93
Coarseness	0.03	Surface (mm^2^)	0.996	RP	0.39
Contrast	0.24	Compacity	0.990		
Busyness	0.04				

Abbreviations: GLNU, gray‐level non‐uniformity; GLCM, gray‐level co‐occurrence matrix; GLRLM, gray‐level run‐length matrix; GLZLM, gray‐level zone length matrix; HGRE, high gray‐level run emphasis; HGZE, high gray‐level zone emphasis; LGRE, low gray‐level run emphasis; LGZE, low gray‐level zone emphasis; LRE, long‐run emphasis; LRHGE, long‐run high gray‐level emphasis; LRLGE, long‐run low gray‐level emphasis; LZE, long‐zone emphasis; LZHGE, long‐zone high gray‐level emphasis; LZLGE, long‐zone low gray‐level emphasis; NGLDM, neighborhood gray‐level difference matrix; Q1, the first quartile; Q2, the second quartile; Q3, the third quartile; RLNU, run‐length non‐uniformity; SRE, short‐run emphasis; SRHGE, short‐run high gray‐level emphasis; SRLGE, short‐run low gray‐level emphasis; SZE, short‐zone emphasis; SZHGE, short‐zone high gray‐level emphasis; SZLGE, short‐zone low gray‐level emphasis; ZLNU, zone length non‐uniformity; ZP, zone percentage.

With the AIIR dataset, 44 features were significantly different (*p* < 0.05) between benign and malignant nodules. These features, therefore, were selected to build the classification model. The same 44 features extracted from HIR images were used to test the applicability. The values of these features between two reconstruction algorithms were compared, and 13.6% (6 of 44) features were influenced by the reconstruction algorithm. The AUCs for these 44 features of two reconstruction algorithm images were shown in Table 3. The entropy of GLCM was with the highest AUC of 0.70 (95% CI, 0.61−0.80) for the AIIR dataset, and not influenced by the reconstruction algorithm (*p* = 0.92). Busyness, one feature from NGLDM, yielded the highest AUC of 0.71(95% CI, 0.60−0.81) with the HIR dataset, and it was influenced by the reconstruction algorithm (*p* = 0.04).

**TABLE 3:** The AUC values for diagnostic performance of features in predicting malignant nodules

	Feature		Feature		Feature	
	**Conventional**	AUC	**Discretized**	AUC	**GLCM**	AUC
	HUmean	0.30^a^	HUmean	0.30^a^	Energy	0.26^a^
	HUstd	0.33^b^	HUstd	0.33^b^	Contrast	0.31^a^
	HUQ1	0.39^b^	HUQ1	0.39^b^	Correlation	0.62^b^
	HUQ2	0.33^b^	HUQ2	0.33^b^	Entropy_log10	0.70^a^
	HUQ3	0.29^a^	HUQ3	0.29^a^	Entropy_log2	0.70^a^
	HUSkewness	0.65^b^	HUSkewness	0.65^b^	Dissimilarity	0.33^b^
	HUKurtosis	0.61^b^	HUKurtosis	0.62^b^	**GLZLM**	
	HUExcessKurtosis	0.61^b^	HUExcessKurtosis	0.62^b^	SZE	0.32^b^
AIIR	**GLRLM**		HISTO_Skewness	0.30^a^	HGZE	0.29^a^
	HGRE	0.30^a^	HISTO_Kurtosis	0.31^a^	SZHGE	0.29^a^
	SRHGE	0.30^a^	HISTO_ExcessKurtosis	0.31^a^	LZHGE	0.34^b^
	LRHGE	0.31^a^	HISTO_Energy	0.39^b^	GLNU	0.68^b^
	GLNU	0.64^b^	**SHAPE**		ZLNU	0.64^b^
	RLNU	0.65^b^	Volume (mL)	0.66^b^		
	**NGLDM**		Volume (vx)	0.65^b^		
	Contrast	0.32^b^	Sphericity	0.39^b^		
	Busyness	0.70^a^	Surface (mm^2^)	0.66^b^		
			Compacity	0.65^b^		
	**CONVENTIONAL**		**DISCRETIZED**		**GLCM**	
	HUmean	0.31^a^	HUmean	0.31^a^	Energy	0.27^a^
	HUstd	0.33^b^	HUstd	0.33^b^	Contrast	0.31^a^
	HUQ1	0.35^b^	HUQ1	0.35^b^	Correlation	0.66^b^
	HUQ2	0.32^b^	HUQ2	0.32^b^	Entropy_log10	0.70^a^
	HUQ3	0.31^a^	HUQ3	0.31^a^	Entropy_log2	0.70^a^
	HUSkewness	0.63^b^	HUSkewness	0.63^b^	Dissimilarity	0.32^b^
	HUKurtosis	0.60^c^	HUKurtosis	0.60^c^	**GLZLM**	
	HUExcessKurtosis	0.60^c^	HUExcessKurtosis	0.60^c^	SZE	0.33^b^
HIR	**GLRLM**		HISTO_Skewness	0.32^b^	HGZE	0.30^a^
	HGRE	0.31^a^	HISTO_Kurtosis	0.31^a^	SZHGE	0.30^a^
	SRHGE	0.31^a^	HISTO_ExcessKurtosis	0.31^a^	LZHGE	0.34^b^
	LRHGE	0.31^a^	HISTO_Energy	0.45^c^	GLNU	0.68^b^
	GLNU	0.65^b^	**SHAPE**		ZLNU	0.65^b^
	RLNU	0.67^b^	Volume (mL)	0.66^b^		
	**NGLDM**		Volume (vx)	0.66^b^		
	Contrast	0.31^a^	Sphericity	0.38^b^		
	Busyness	0.71^a^	Surface (mm^2^)	0.66^b^		
			Compacity	0.65^b^		

Abbreviations: GLNU, gray‐level non‐uniformity; GLCM, gray‐level co‐occurrence matrix; GLRLM, gray‐level run‐length matrix; GLZLM, gray‐level zone length matrix; HGRE, high gray‐level run emphasis; HGZE, high gray‐level zone emphasis; LGRE, low gray‐level run emphasis; LGZE, low gray‐level zone emphasis; LRE, long‐run emphasis; LRHGE, long‐run high gray‐level emphasis; LRLGE, long‐run low gray‐level emphasis; LZE, long‐zone emphasis; LZHGE, long‐zone high gray‐level emphasis; LZLGE, long‐zone low gray‐level emphasis; NGLDM, neighborhood gray‐level difference matrix; Q1, the first quartile; Q2, the second quartile; Q3, the third quartile; RLNU, run‐length non‐uniformity; SRE, short‐run emphasis; SRHGE, short‐run high gray‐level emphasis; SRLGE, short‐run low gray‐level emphasis; SZE, short‐zone emphasis; SZHGE, short‐zone high gray‐level emphasis; SZLGE, short‐zone low gray‐level emphasis; ZLNU, zone length non‐uniformity; ZP, zone percentage.

^a^ *p* < 0.001.

^b^ *p* < 0.05.

^c^ Not significant.

### 3.2 Classification model

For the features extracted from AIIR images, multiple combinations were used to train and test the model. Finally, 44 features with significant differences between benign and malignant nodules were used to train and test the model. The diagnostic ability of the classification model was evaluated by comparing the accuracy in discriminating benign and malignant nodules. As found by the LOOCV, the diagnostic accuracy was 98.3%, and the AUC value was 0.98 (95% CI, 0.95−1.00). The sensitivity, specificity, PPV, and NPV of the classification model were 96.2%, 100.0%, 100.0%, and 97.1%, respectively.

With the validation set, the diagnostic accuracy of the model was found to be 92.3%, and the AUC value was 0.91 (95% CI, 0.74−0.90). The sensitivity, specificity, PPV, and NPV of the classification model were 93.8%, 90.0%, 93.8%, and 90.0%, respectively.

**FIGURE 4:**
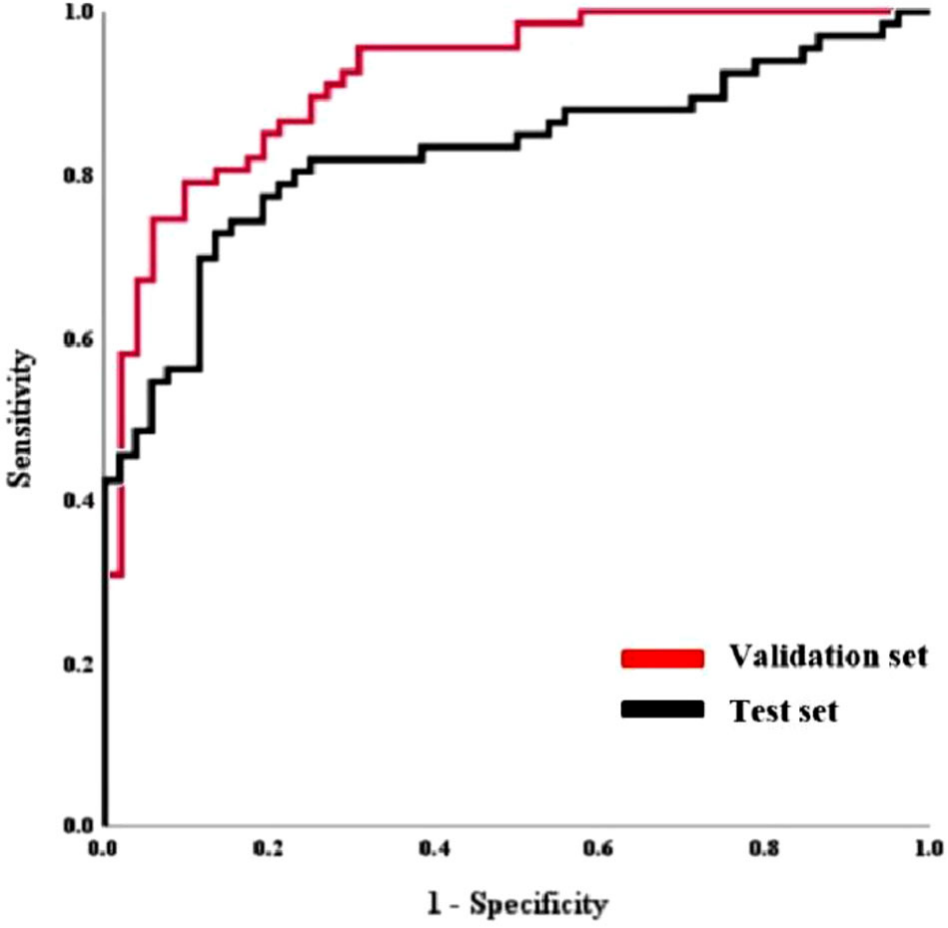
Receiver operating characteristic curve (ROC) analysis of the classification model with the validation and the test set. The areas under the ROC curve (AUCs) are 0.91 (95% confidence interval [CI], 0.74−0.90) and 0.80 (95% CI, 0.68−0.86) for validation and test, respectively.

With the test set, that is, the HIR data, the accuracy and AUC were 80.0% and 0.80 (95% CI, 0.68−0.86), respectively, which were lower than those found in the validation of the model itself. The sensitivity, specificity, PPV, and NPV were 89.4%, 67.3%, 77.6%, and 83.3%, respectively. Figure 4 shows the ROC curves. Based on such results, the classification model trained from AIIR dataset seems to be applicable to the HIR dataset as input.

## 4 DISCUSSION

This research was the first to investigate the applicability of the CT texture analysis model between the generations of reconstruction algorithms, whereas, in most other studies, researchers have been focused only on the benefit of the advanced reconstruction algorithms to the classification model.^32^, ^33^ As confirmed by this study, the texture analysis model derived from AIIR images preformed the best in classifying pulmonary nodules and showed the highest AUC values. Of more interest, we also found that such a model was in general applicable to HIR images as the input, with reduced accuracy though. Such finding suggests the potentially wider applicability of the CT texture analysis models, which is in particular beneficial to settings where the latest‐generation technology remains yet accessible.

Texture features were influenced by CT reconstruction algorithms in our study, which was consistent with those published by previous researchers.^34^, ^35^ Meyer et al. reported that only 11.3% features were reproducible for different reconstruction parameters.^36^ However, 75.0% texture features were reproducible between the latest‐ and last‐generation reconstruction images in our study. The result showed that AIIR may have the ability to improve the reproducibility of texture analysis features between AIIR and HIR algorithm.

A previous study demonstrated that the radiomics model in diagnosing the invasiveness of lung adenocarcinoma yielded an AUC of 0.98 and accuracy of 93%.^9^ Our classification model derived from AIIR images yielded an AUC value of 0.91 and accuracy of 92.3% in its test for backward applicability, which was excellent. This is not difficult to explain because most texture features input into the classification model were significantly different between benign and malignant nodules in either AIIR or HIR images. However, 13.6% features selected into the model were influenced significantly in the switch of reconstruction algorithm, which caused the decrease in diagnostic accuracy.

Texture features associated with the heterogeneity of tumor have been reported as important prognosticators.^33^ Busyness, one feature of NGLDM, was influenced by the reconstruction algorithm significantly and played an important role in the classification model with the highest AUC of 0.71 in HIR dataset. Several previous studies also reported that busyness showed high potential as an imaging biomarker in pathological diagnosis.^37^, ^38^ GLCM features have been used as the traditional imaging markers in the pulmonary nodules segmentation and outcome prediction.^39^, ^40^ In our study, the entropy of GLCM showed highest AUC of 0.70 in AIIR dataset for the differentiation between benign and malignant nodules, and this feature was not influenced by the changes of reconstruction algorithm.

To investigate the effect of the correlation that several nodules were from the same patients’ sets, the data in our study were re‐screened, and only one nodule per patient was included in the training and testing datasets. After the classification model re‐training and re‐testing, the AUC based on AIIR images was 0.9 and 0.78 when performing on HIR images. Beig et al. reported that the AUC of the convolutional neural network model was 0.76 for distinguishing adenocarcinomas from granulomas.^3^ Another radiomics‐based model to predict lymph node status was with the AUC of 0.77.^7^ The AUCs in our study were higher than those in previous studies and acceptable in clinical research.

The method used to create the classification model is a very important part, which can determine the accuracy of the classification model to a large extent. In recent years, with the application of machine learning in medical image processing, more and more researchers use machine learning methods to build classification models. Traditionally, researchers mostly use Cox and binary regression analysis to establish classification models and have obtained good results.^41^, ^42^ Ohno et al. reported that texture analysis based on machine learning has the potential to improve the accuracy of diagnosis and interobserver agreement in lung diseases.^43^ Another study showed that an artificial neural networks model for differentiation of preinvasive lesions from invasive pulmonary adenocarcinoma yielded AUC of 0.981.^44^ The neural network‐based texture analysis model in our study showed pretty high diagnostic accuracy and AUC. To improve the diagnostic performance of classification models, a more powerful algorithm should be investigated in further research.

There are several limitations in our study. Firstly, all malignant lesions in this study were adenocarcinoma, which may have some impacts on the specificity of the classification model. Secondly, this study only carried out texture analysis classification model for predicting benign and malignant pulmonary nodules. In the future, data from multiple body parts shall be included to investigate the applicability for other pathologies. Thirdly, the raw‐data reconstructed using AIIR and HIR algorithm were from the same patients sets. In the further research, it was with significant clinical value to train and test the classification model using data from two different sets of patients.

## 5 CONCLUSION

In context of the relatively mature and common task of using CT texture analysis to discriminate benign and malignant pulmonary nodules, a model trained with the latest‐generation deep‐learning reconstruction (AIIR) images has certain applicability to the last‐generation iterative reconstruction (HIR) images. Caution is advised, though, for a decrease of accuracy.

## CONFLICT OF INTEREST

The authors declare that they have no conflict of interest.

## AUTHOR CONTRIBUTIONS

Qingle Wang and Shijie Xu contributed to the acquisition, analysis, interpretation data, and draft for the work. Guozhi Zhang and Xingwei Zhang contributed to the revising of the important intellectual content for the work. Junying Gu and Shuyi Yang contributed to the interpretation of data for the work. Mengsu Zeng and Zhiyong Zhang contributed to the conception and design of the work. All authors contributed to the final approval of the version to be published, agreement to be accountable for all aspects of the work in ensuring that questions related to the accuracy or integrity of any part of the work were appropriately investigated and resolved.
